# Supplementary material for: Polyketide synthase gene clusters in the ericoid mycorrhizal fungus Oidiodendron maius: genome-wide prediction, expression profiling, and functional characterization of knockout mutants
Source: Front Plant Sci. 2026 Sep 16;17:1924430. doi: 10.3389/fpls.2026.1924430 (PMC13623903; doi:10.3389/fpls.2026.1924430)
Supplement: Supplementary file 1 [file SupplementaryFile1.zip › Supplementary Materials/Supplementary Folder 1.zip!/Oidma1_AssemblyScaffolds_Repeatmasked/knownclusterblast/cluster50/jgi.p_Oidma1_45995_mibig_hits.html]

| MIBiG Protein | Description | MIBiG Cluster | MiBiG Product | % ID | % Coverage | BLAST Score | E-value |
| --- | --- | --- | --- | --- | --- | --- | --- |
| CAA60450.1 |  | BGC0001040 | Rapamycin\_biosynthetic\_gene\_cluster | 47 | 97.7777777778 | 358 | 1.1e-98 |
| ACZ65471.1 | putative\_dipeptidyl\_aminopeptidase | BGC0000140 | RK-682\_biosynthetic\_gene\_cluster | 49 | 62.2222222222 | 239 | 5.8e-63 |
| ACS68553.1 | alpha/beta\_hydrolase | BGC0001026 | NG-391\_biosynthetic\_gene\_cluster | 33 | 102.716049383 | 210 | 4.9e-54 |
| EAL85114.1 | alpha/beta\_hydrolase,\_putative | BGC0001037 | Pseurotin\_A\_biosynthetic\_gene\_cluster | 34 | 88.8888888889 | 166 | 6.2e-41 |
| EAU38973.1 | predicted\_protein | BGC0001122 | Isoflavipucine\_biosynthetic\_gene\_cluster | 33 | 88.6419753086 | 156 | 4.9e-38 |
| EAW09119.1 | conserved\_hypothetical\_protein | BGC0000983 | Cytochalasin\_biosynthetic\_gene\_cluster | 35 | 84.1975308642 | 152 | 9.3e-37 |
